# *In vitro* study of resveratrol as an antioxidant for boar semen preservation: A systematic review

**DOI:** 10.14202/vetworld.2025.85-94

**Published:** 2025-01-14

**Authors:** Ragil Angga Prastiya, Trilas Sardjito, Amung Logam Saputro, Sari Yanti Hayanti, Nining Haryuni, Samira Musa Sasi

**Affiliations:** 1 Department of Health, Medicine and Life Sciences, Faculty of Health, Medicine and Life Sciences, Universitas Airlangga, Banyuwangi, 68423, Indonesia; 2 Center for Animal Husbandry, Research Organization for Agriculture and Food, National Research and Innovation Agency of The Republic of Indonesia, Cibinong Sciences Center, Cibinong, Bogor, 16911, Indonesia; 3 Department of Animal Production, Animal Husbandry Study Program, Nahdlatul Ulama Blitar University, Blitar, Indonesia; 4 Department of Zoology, Faculty of Science, University of Tripoli, Tripoli, Libya

**Keywords:** antioxidant, artificial insemination, boar semen preservation, livestock and gene bank, resveratrol

## Abstract

**Background and Aim:**

In the global pig farming industry, artificial insemination (AI) is widely used to propagate high-quality genetics using processed semen that can be stored for extended periods. However, boar sperm are particularly susceptible to oxidative damage during storage and cryopreservation because of their high polyunsaturated fatty acid content. This study aimed to systematically review the impact of resveratrol (RVT), a potent antioxidant, on the preservation of boar semen.

**Materials and Methods:**

A comprehensive meta-analysis was conducted following the Preferred Reporting Items for Systematic Reviews and Meta-Analyses guidelines. Relevant articles were retrieved from databases such as Scopus, ScienceDirect, and PubMed using specific keywords. After a thorough screening, 10 studies were selected for inclusion. Data extracted from these studies included parameters such as sperm motility, viability, DNA integrity, and lipid peroxidation levels.

**Results:**

Resveratrol supplementation in boar semen extenders significantly improved sperm quality under various storage conditions, including waterbath and cooled and frozen semen. RVT’s antioxidative properties effectively reduced reactive oxygen species and prevented oxidative stress-related damage to sperm cells.

**Conclusion:**

The addition of resveratrol to semen extenders enhances the preservation of boar sperm by mitigating oxidative damage, potentially increasing the success rate of AI in the swine industry. This study highlights the need for further research to optimize RVT dosage and application methods for different semen preservation techniques.

## INTRODUCTION

In pig farms worldwide, artificial insemination (AI) has become a program for accelerating and disseminating high-quality genetics using processed semen that can be stored for an extended period [1]. Commonly, boar semen is stored at temperatures of 15°C–20°C for up to 5 days. To ensure long-term preservation of the quality of boar sperm, their metabolic activity should be reduced by storing semen at low temperatures (cryopreservation) and adding an appropriate extender media [2]. Cryopreservation techniques, particularly in boars, have rapidly developed, focusing on selecting preservation materials or media, optimizing frozen semen storage, and assessing boar sperm quality before and after freezing [3].

The membrane of boar sperm is rich in polyunsaturated fatty acids, making it particularly vulnerable to oxygen-induced lipid peroxidation [4]. Boar sperm experience cold shock during cryopreservation, which results in harmful cellular alterations mostly because of elevated reactive oxygen species (ROS) levels, which include superoxide anions, hydroxyl radicals, and hydrogen peroxide. These ROS are generated during the intermediate stages of oxygen reduction and can damage DNA, plasma membrane lipids, and cellular proteins [5]. Although low and controlled ROS levels are essential for sperm functions, such as hyperactivation, capacitation, acrosome reaction, and zona binding, excessive ROS production impairs the sperm’s ability to adapt, resulting in oxidative stress and cellular damage [6]. Consequently, thawed sperm may exhibit structural changes in nucleoprotein-DNA and alterations similar to capacitation, significantly diminishing their fertilizing capacity [7]. To mitigate oxidative damage, enhancing the semen extender with both enzymatic and non-enzymatic antioxidants during the freezing and thawing processes is a recommended approach [8].

Antioxidants have been added to boar semen extenders to prevent membrane permeabilization, retain membrane integrity (MI), and lower lipoperoxidation to lessen oxidative stress. Boar semen contains compounds that have been used to treat oxidative stress, including glutathione, α-tocopherol, catalase, L-glutamine, and superoxide dismutase [9, 10]. Resveratrol (3,5,4’-trihydroxystilbene, a stilbenoid; RVT), a naturally occurring polyphenolic substance generated in response to environmental stressors, is another commonly used antioxidant in boar semen [11]. RVT shares structural similarities with estradiol and diethylstilbestrol. Originally, *Polygonum cuspidatum*, a plant utilized in traditional Chinese medicine for its healing qualities against wounds, stress, bacterial and fungal infections, UV radiation, and ozone exposure, was found to have the majority of RVT in its dried roots [12, 13]. Resveratrol is known to possess antioxidant properties in somatic cells, such as reducing reactive oxygen species (ROS) production in the mitochondria, counteracting superoxide radicals, preventing lipid peroxidation, and regulating the expression of antioxidant cofactors and enzymes [14].

RVT seems more effective in scavenging reactive oxygen species (ROS) rather than preventing radical generation. The lipophilic nature of RVT enables its penetration into the plasma membrane, allowing its antioxidant properties to function in both intracellular and extracellular settings. RVT has been used to improve the quality of liquid or chilled semen in bulls [15–17], rams [18], and boars [1, 19, 20]. Furthermore, it has been incorporated into frozen or thawed semen media for human applications [21]. A recent study found that a concentration of 40 μg/mL of nano-encapsulated RVT in the extender improved the kinematic patterns of post-thawed wild boar sperm [22].

This study aimed to compile all available information regarding the inclusion of RVT in boar semen extenders and present it through a meta-analysis, providing a statistically quantifiable overview of the beneficial effects of RVT.

## MATERIALS AND METHODS

### Ethical approval

This meta-analysis does not require ethics approval from the ethics committee because it does not involve any laboratory or animal subjects. The study is based exclusively on a review of the existing literature. Peer-reviewed articles were carefully selected and evaluated in compliance with the guidelines set out by Preferred Reporting Items for Systematic Reviews and Meta-Analyses 2020 [23].

### Study period and location

This study was conducted from February to December 2023. Data extraction and analysis were performed at Faculty of Health, Medicine, and Life Sciences, Universitas Airlangga.

### Search strategy

The search for relevant published articles was conducted using various databases, including Scopus (https://www.scopus.com/home.uri), ScienceDirect (http://www.sciencedirect.com/), and PubMed (https://www.ncbi.nlm.nih.gov/pubmed), with specific search terms such as “Resveratrol,” “Phytoalexin,” “3,5,4’-trihydroxystilbene,” “antioxidant,” “boar,” “pig,” “extender,” “sperm,” “cryopreservation,” and “frozen.” After importing all the titles generated from each search, duplicates were carefully eliminated to ensure the selection of eligible studies. This search was conducted in December 2023 without any restrictions on the number of years of publication.

### Study eligibility

**Figure 1:**
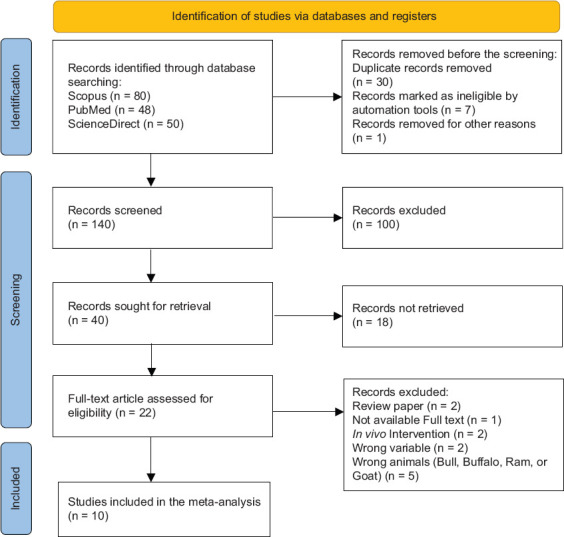
Diagram flow of article selection in the meta-analysis using Preferred Reporting Items for Systematic Reviews and Meta-Analyses method.

Journal articles published in English met the main inclusion criteria. The experimental designs had to adhere to established statistical principles, and the materials needed for the experiments and replications had to meet the requisite statistical standards. The number of boars used (n) required to meet the necessary statistical standards, and the study had to incorporate experimental animal materials of boar. Figure 1 shows the inclusion and exclusion criteria of the articles.

### Data extraction and selection

Author(s) names, journal name, publication year, variety (breed) of boars, number of boars, Resveratrol and its analog dose, study parameters, suggested extricated dose, type of Resveratrol (e.g., 3,5,4′-trimethoxystilbene, triacetyl-resveratrol, and 3,5,4’- trihydroxystilbene), units for every parameter, inspecting method, and parameter measurement technique were extracted from the selected articles. Full-text articles were submitted to a Mendeley reference manager, and four authors screened the titles and abstracts to assess the published articles. The search identified 140 articles depicting investigations of resveratrol supplementation in extenders. Notwithstanding, considering their titles and abstracts, only 22 articles were included.

After a comprehensive evaluation, 10 eligible articless were included in the metadata: Sun *et al*. [2]; Martín-Hidalgo *et al*. [1]; Bucci *et al*. [20]; Gadani *et al*. [19]; Soares *et al*. [22]; Prommi *et al*. [24]; He *et al*. [25]; Torres *et al*. [26]; Zhu *et al*. [27]; and Kaeoket and Chanapiwat [28]. The selected articles included studies on chilling and frozen semen. Sperm motion using a sperm analyzer (computer-assisted sperm analysis; CASA), acrosome damage (%), acrosomal integrity (%), DNA damage (%), DNA integrity (%), malondialdehyde (MDA) content (nmol/mL), sperm motion (%), sperm viability (%), progressive motility (PM) (%), and total motility (TM) (%) were assessed to determine how resveratrol supplementation affected the extender. The process of studies selection is mentioned in Figure 1.

### Quality criteria

After the final selection of the articles, a good quality methodological analysis was conducted based on the set by 24. The following parameters were used:

1. Experiments with 5 or more boars earned two points, whereas those with fewer than 5 earned one.
2. Replications/treatments: Those reporting five or more points per treatment receive three points; those testing less than five receive two, and the rest receive one.
3. Boar age: Those reporting and matching the age of sows score two points, those not matching or not reporting this factor one.
4. Variables: Studies that measured sperm quality characteristics and apoptotic parameters (AP) received two points; studies that did not measure AP received one point.
5. Sperm collection: Studies that include details of sperm collection performed by a trained person or procedure approval, method, and collection frequency received two points. Those describing three details receive two points, and those describing one detail or that is not mentioned received one point.
6. Extender type: Studies that mention the type of extender used and the composition received two points, whereas studies where it is not mentioned or the mention is not satisfactory received one point.
7. Resveratrol composition: Studies showing the use or absence of resveratrol and the type of resveratrol in the diluter receive two points. Studies that do not mention this factor received one point.
8. Randomization: When experimental randomization is clearly described, it received two points; otherwise, if no randomization or its unclear description is mentioned in the text, it received one point.
9. Studies that analyzed sperm immediately after collection received two points for their fresh analysis. One point was awarded to studies that did not analyze fresh sperm.
10. Methodology: CASA for testing acrosome integrity (AP) received three points; those using either CASA or AP testing received two points; and those that did not mention either received one point.
11. Preservation method: Experiments explaining frozen or chilled preservation methods received two points, if not mentioned, received one point.
12. Dose recommendations: Experiments explaining and concluding the recommended doses to be used received two points; if not mentioned, received one point.

## RESULTS

Table 1 provides an overview of the key dataset and descriptive statistics related to the articles. The results of the research from the 10 articles we collected, which are relevant to the research topic, are presented in Table 2. The information required for our data analysis is presented in Table 2. Based on the predetermined criteria, Kaeoket and Chanapiwat [28] and Torres *et al*. [26] received the highest score of 26. The second-ranking paper was Soares *et al*. [22] with 25 points, followed by Sun *et al*. [2] with 24 points. The study by Gadani *et al*. [19] received the lowest score of 19. None of the research articles reached an optimal score of 27 points because many articles lacked explanations about sample randomization and did not provide recommended dosages of resveratrol that could be implemented in the conclusions.

**Table-1:** Studies included in the systematic review of the effect of resveratrol supplementation on the sperm quality assessment of boars.

References	1	2	3	4	5	6	7	8	9	10
[1]	6	2	DB; PB and LW	24 – 48	BTS and DM	3,4′,5-Trihydroxy-trans-stilbene	0; 10; 33; 66 and 100 μM	WB	7 days	SP
[2]	10	4	DB	20	ME	Resveratrol (R5010; Sigma-Aldrich)	0; 25; 50; 100; 150 and 200 μM	CS and WB	5 days	SP; AP
[19]	3	3	LW	24 – 48	BTS	Resveratrol (R5010; Sigma-Aldrich)	0; 0.5; 1; 2 mM	WB	1 hours	SP
[20]	3	6	LW	24 – 48	BTS	Resveratrol (R5010; Sigma-Aldrich)	0 mM; 2 mM; 2 mM+50 μM EGCG	WB	1 and 4 hours	SP; AP
[22]	5	7	Boar	24 – 48	BTS	Resveratrol (R5010; Sigma-Aldrich) and Resveratrol encapsulated	0; 5; 10; 20; 40; 80 μg/mL	LN	1 hours	SP; AP
[24]	9	9	DB; PB	12 – 36	ME	Oxyresveratrol	0; 3.125; 6.25; 12.5; 25 μM	LN	NI	SP
[25]	3	4	MP	24 – 48	TS	Resveratrol (R5010; Sigma-Aldrich)	0 and 1 mmol/L	LN	NI	SP; AP
[26]	6	6	Boar	24 – 48	BTS	Resveratrol (R5010; Sigma-Aldrich)	0; 0.01; 0.1; 1 mM	WB	3 days	SP; AP
[27]	21	21	DB	24	ME	Resveratrol (R5010; Sigma-Aldrich)	0; 25; 50; 75; 100; 125 μM	LN and CS	NI	SP; AP
[28]	13	1	DB	18 – 36	ME	Resveratrol (R5010; Sigma-Aldrich)	0, 25, 50, 75, 100, 125, and 250 μM	LN	10 years	SP; AP

1) Total of boars in the study; 2) Number of repetitions per treatment; 3) Boar breed (DB- Duroc Boar; MP- Meishan Pigs; PB-Pietrains Boar; LW- Large White; 4) Age (month); 5) Type of Extender (BTS- Beltsville Thawing Solution; ME- Modena Extender; TS- TCG solution; DM- DMSO 14%); 6) Type of Resveratrol; 7) Dosage; 8) Preservation method (LN- liq nitrogen storage; CS- refrigerated (4-5 °C); WB – water bath (17 °C)); 9) Storing time; 10) Variables (SP- Sperm quality parameters; AP – Apoptotic parameters). NI- not informed.

**Table 2:** Evaluation criteria scores for the selected articles in the systematic review *in vitro* study of resveratrol as an antioxidant in boar semen preservation

Author	1	2	3	4	5	6	7	8	9	10	11	12	Total
Martin-Hidalgo et al. [1]	2	2	2	1	2	2	2	2	2	2	2	1	22
Sun et al. [2]	2	2	2	2	2	2	2	1	2	3	2	2	24
Gadani et al. [19]	1	2	2	1	1	2	2	1	1	2	2	2	19
Bucci et al. [20]	1	3	2	2	1	2	2	1	1	3	2	1	20
Zhu et al. (2019)	2	3	2	2	3	2	2	1	1	3	2	1	22
Soares et al. [22]	2	3	2	2	2	2	2	1	2	3	2	2	25
Prommi et al. [24]	2	3	2	1	2	2	2	1	2	2	2	2	23
He et al. [25]	1	2	2	2	2	2	2	1	2	3	2	1	22
Torres et al. [26]	2	3	2	2	2	2	2	2	2	3	2	2	26
Kaeoket and Chanapiwat [28]	2	2	2	2	3	2	2	2	2	3	2	2	26

1. Total number of boars used: Experiments using 5 or more boars (2); those using less than 5 boars (1).

2. Number of repetitions/treatments: Experiments with 5 or more repetitions/treatment (3); less than 5 receive (2); and not mentioned (1).

3. Age of boars: Experiments describing and matching the age of boars (2); those not matching or not reporting this (1).

4. Variables: Experiments evaluating sperm quality characteristics and apoptotic parameters (2); not evaluating apoptotic parameters (1).

5. Sperm collection: Experiments providing all details of sperm collection by a trained person or procedure approved, method, and frequency of collection (3); describing two details (2), and describing one details or not mentioned (1).

6. Extender type: Experiments describing the type of extender used along with its composition receive (2), and not explaining (1).

7. Resveratrol composition: Experiments mentioning the use and the type of resveratrol in the diluter (2); not mentioned (1).

8. Randomization: Experiments clearly describing the randomization of the experiment (2), and those that are not randomized (1).

9. Fresh sperm analysis: Experiments evaluating sperm immediately after collection (2); those not evaluating fresh sperm (1).

10. Methodology: Experiments using computer-assisted sperm analysis (CASA) and equipment for testing apoptotic parameters for sperm analysis (3); those using either of these techniques (2), and those not mentioning (1).

11. Preservation method: Experiments explaining frozen or chilled preservation methods (2), not mentioned (1).

12. Dose recommendations: Experiments explaining and concluding the recommended doses to be used (2), not mentioned (1)

From the results summarized in Table 1, we identified that 100% of the articles mentioned the age of the boar used as a semen donor, the type of extender used, the composition of resveratrol used, and the preservation methods applied in the research. In addition, the summary of results indicated that 70% of the articles explained the details of the total number of boars used, variables related to spermatozoa quality and apoptosis tests, assessments of fresh semen viability, and specific laboratory testing methods employed. Only 60% of the articles elaborated on the total repetition of treatment and recommended resveratrol dosages. Only the study by Zhu *et al*. [27] and Kaeoket and Chanapiwat [28] provided comprehensive information about conducted experiments outlining the complete process of sperm retrieval, carried out by a certified individual or an endorsed procedure, including the technique and regularity of collection. Among the articles, 70% explained the randomization of the experiments.

Nearly, all articles used the resveratrol type R5010 from Sigma-Aldrich. However, Martin-Hidalgo *et al*. [1] used 3,4′,5-trihydroxy-trans-stilbene. In addition, Soares *et al*. [22] explored encapsulated resveratrol in combination with resveratrol R5010. Prommi *et al*. [24] used oxyresveratrol. There was no consistency in the dosage of resveratrol across the articles, with only Kaeoket and Chanapiwat [28], Sun *et al*. [2], and Zhu *et al*. [27] using the same treatment doses: 0, 25, 50, and 100 μM, although the final doses differed.

Torres *et al*. [26] did not specify the breed of boar used. The other articles consistently detailed the breed of the boar. The most frequently used breeds were Duroc and Large White, with Pietrains and Meishans also being used. The Meishan pig originates from the Meishan region in Sichuan Province, China, while the Pietrain pig originates from the Ardennes region in Belgium. The age of the boar ranged from 20 to 48 months, which was explicitly described in the methodology of all the articles.

In this study, the most frequently used diluents were Beltsville Thawing Solution (BTS) and Modena Solution. BTS is a product manufactured by Minitüb GmbH, Tiefenbach, Germany. Modena Solution is composed of 46.6 mM Tris, 15.1 mM citric acid, 11.9 mM sodium hydrogen carbonate, 26.7 mM trisodium citrate, 6.3 mM EDTA-2Na, 153 mM D-glucose, 1000 IU/mL penicillin G sodium salt, 100 μg/mL polymyxin B, and 1 mg/mL streptomycin sesquisulfide. In addition, other extenders mentioned in the reviewed articles include 14% dimethyl sulfoxide and a Tris-citric-glucose solution, which contains 2.42 g Tris, 1.10 g glucose, 1.48 g citric acid, and 100,000 IU of penicillin-streptomycin solutions.

### Characterization of the main results

#### Water bath semen

Research on the use of resveratrol in semen storage using the water bath method (17°C) revealed varying results. Sun *et al*. [2] and Zhu *et al*. [27] reported the most significant outcomes. Sun *et al*. [2] reported that resveratrol concentrations of 25–200 μM led to increases in TM and PM. They also reported improvements in MI, acrosome integrity (AI), and total antioxidant capacity (T-AOC). Zhu *et al*. [27] corroborated these results, showing significant increases in TM and PM with resveratrol concentrations of 25–125 μM. Improvements in sperm viability, MI, and T-AOC were also observed, indicating that resveratrol effectively preserves sperm quality during water bath storage. However, Torres *et al*. [26] reported contrasting results, with decreases in all tested parameters. In this study, resveratrol concentrations of 0.01–1 mM did not improve TM, PM, or other sperm quality parameters (SP). Similar findings were reported by Bucci *et al*. [20] and Gadani *et al*. [19], who reported that resveratrol did not significantly improve the tested SP. Martin-Hidalgo *et al*. [1] also reported either a decline or no significant improvement in TM and PM with resveratrol use. The MI and viability (V) did not significantly change.

#### Cooled semen

An increase in sperm motility was observed in most studies using resveratrol as an antioxidant for semen preservation. Sun *et al*. [2] and Zhu *et al*. [27] reported the most significant results. Sun *et al*. [2]reported improvements in TM and PM with resveratrol concentrations ranging from 25 to 200 μM. In addition, they observed enhancements in various SP, including MI, AI, and sperm mitochondrial membrane potential. Zhu *et al*. [27] supported these findings, with results showing significant increases in TM and PM when using resveratrol concentrations of 25–125 μM. Sperm viability also improved significantly, and parameters such as MI and T-AOC showed positive outcomes. This indicates that resveratrol effectively maintains sperm quality during cooled semen storage.

#### Frozen semen

An increase in sperm motility was observed in all studies evaluating frozen semen, except for Torres *et al*. [26], which reported a decrease. Kaeoket and Chanapiwat [28] demonstrated significant increases in TM and PM using resveratrol concentrations of 0–250 μM. They noted significant improvements in SP and AP, indicating that resveratrol can reduce oxidative damage and enhance sperm viability. Soares *et al*. [22] also found significant increases in TM, PM, and MI with the use of resveratrol and encapsulated resveratrol at concentrations of 5–80 μg/mL. These findings suggest that resveratrol effectively maintains sperm quality during freezing and storage. He *et al*. [25] showed that resveratrol at a concentration of 1 mmol/L improved MI and reduced AP, indicating that resveratrol effectively mitigates oxidative damage in frozen sperm. Zhu *et al*. [27] reported significant improvements in TM, PM, and sperm viability at resveratrol concentrations of 25–125 μM. They also reported enhancements in MI and T-AOC, demonstrating resveratrol’s efficacy in reducing oxidative damage during the freezing and storage of semen. Prommi *et al*. [24] reported that the use of oxyresveratrol at concentrations of 3.125–25 μM increased TM, PM, and other SP. These findings indicate that oxyresveratrol is effective in maintaining sperm quality during freezing and storage.

Overall, resveratrol positively affects boar semen quality during water bathing and freezing storage. Parameters such as sperm motility, viability, and MI tend to improve with the use of resveratrol at various concentrations and storage methods (Table 3). The varied results among the studies may be due to differences in study design, resveratrol concentrations, type of extender used, and semen storage methods.

**Table 3:** Main selected results articles in the systematic review *in vitro* study of resveratrol as an antioxidant in boar semen preservation.

Author	Parameters^c^																			

	TM	PM	MI	AI	MMP	V	KF	DF	ATPc	MDA	TAR	AS	LP	TBARS	ROS	SOD	GSH	GPx	Cat	T-AOC
Martin-Hidalgo et al. [1]	-	NS	NP	+	-	NS	-	NP	-	NP	NP	NP	NP	NP	NP	NP	NP	NP	NP	NP
Sun et al. [2]	+	+	+	+	+	NP	NP	NP	NP	-	NP	NP	NP	NP	-	NP	NP	NP	NP	+
Gadani et al. [19]	NP	NP	NP	NS	NP	NS	NP	NP	NP	NP	NP	NP	NP	NP	NP	NP	NP	NP	NP	NP
Bucci et al. [20]	-	-	NP	NS	NP	NS	NP	+	NP	NP	NP	NP	NS	NP	NP	NP	NP	NP	NP	NP
Soares et al. [22]	+	+	+	+	+	NP	NS	-	NP	NP	NP	NP	+	NP	NP	+ (RVT) - (RVT-en)	NP	NP	NP	NP
Prommi et al. [24]	NP	+	NP	NS	NP	+	NP	NP	NP	NP	NP	NP	NP	NP	NP	NP	NP	NP	NP	NP
He et al. [25]	NS	NP	+	+	+	NP	NP	NP	+	NP	-	NP	NP	NP	-	NP	NP	NP	NP	NP
Torres et al. [26]	-	-	-	-	NS	NP	-	NP	NP	NP	NP	NP	NP	+	NP	-	NP	NP	NP	NP
Zhu et al. [27]	+	+	+	+	+	NP	+	NP	NP	NP	NP	v	-	NP	-	+	+	+	+	NP

^a^Semen type (LN: liquid nitrogen; CS: cooled semen 4-5°C; WB: water bath at 17°C).

^b^Type of Resveratrol (RVT: resveratrol; ORVT: oxyresveratrol; RVT-en: resveratrol encapsulated; TS: 3,4′,5-Trihydroxy-trans-stilbene)

c Parameters (NP: not provided. NS: not significant. +: increased in some treatments. -: decreased in some treatments)

TM: Total Motility; PM: Progressive Motility; MI: Membrane Integrity; AI: Acrosome Integrity; MMP: Sperm mitochondrial membrane potentials;

V: Viability; KF: Kinematic Features; DF: DNA Fragmentation; ATPc: ATP content; MDA: Malondialdehyde; TAR: TUNEL Apoptotic rate; AS: Apoptotic sperm; LP: Lipid Peroxidation; TBARS: Thiobarbituric Acid Reactive Substances; ROS: Reactive oxygen species; SOD: Superoxide Dismutase;

GSH: Glutathione; GPx: Glutathione peroxidase; Cat: Catalase; T-AOC: Total Antioxidant Capacity

## DISCUSSION

Supplementation of boar sperm with antioxidants is a technique mainly involved in the preservation of boar sperm quality to increase fertilization capacity after storage and reduce oxidative stress [29]. Pre-storage processing time reduces antioxidant concentrations in seminal plasma and spermatozoa, and this reduction is almost always related to either oxidative or nitrosative stress [30]. Excessive ROS/RNS production can overwhelm the antioxidant defense system and send the cell into a harmful condition known as oxidative/nitrosative stress, which upsets the homeostatic balance between ROS/RNS and antioxidant production. This process may harm sperm biomolecules, including lipids, nucleic acids, and proteins, resulting in modifications of their ability to move and fertilize [31, 32]. Excessive production of ROS can increase lipid peroxidation of sperm membranes, reducing sperm capacitance, causing a decrease in sperm motility, and lowering sperm viability, thereby reducing fertility [25, 33].

Antioxidants play an important role as scavengers of ROS and RNS. They do not induce oxidative damage to the sperm cells. Antioxidants must be added to semen diluters during cooling and freezing procedures when the level of oxidative stress is high [34, 35]. Different types of antioxidants have been studied that significantly improved sperm motility, viability, and quality during storage [36]. These include but are not limited to, vitamin C, vitamin E, and glutathione. Antioxidants in semen diluters are important, particularly during cooling or freezing processes, because the action of these oxidants is very highly expressed. Antioxidants added to diluters ensure that sperm membranes remain intact, DNA is protected from oxidative attack, and mitochondrial function is maintained (responsible for sperm motility and energy generation) [37].

Resveratrol, a natural polyphenolic compound found in various plants such as grapes and berries, has been widely studied for its antioxidant properties in different animal species [2]. The agent has shown promising results in improving sperm quality, motility, and fertility in mice, rats, and cattle. However, the potential of resveratrol in boar semen preservation remains relatively underexplored [27]. While several studies [6-8] have demonstrated the efficacy of this agent in enhancing sperm quality and reducing oxidative stress in other animals, there is a need for more focused research on its application in boars. Understanding the specific effects and mechanisms of resveratrol in boar semen could provide valuable insights into developing more effective preservation methods.

Resveratrol offers several benefits for boar semen preservation. It acts as a potent antioxidant, scavenging free radicals, and reducing oxidative damage to sperm cells. In addition, resveratrol has anti-inflammatory properties that help mitigate inflammation-related damage during storage [38]. The ability of sperm to enhance mitochondrial function and protect MI contributes to improved sperm motility and viability [1]. Resveratrol also exhibits anti-apoptotic effects, reducing programmed cell death in sperm cells and maintaining their fertilizing potential. By incorporating resveratrol into semen preservation protocols, it is possible to improve the quality and longevity of stored boar sperm, ultimately enhancing reproductive outcomes [27].

Using resveratrol with nano-encapsulation for cryopreserved semen can enhance water solubility and maintain stable temperature and pH levels for antioxidants [39]. Consequently, these antioxidants exhibited increased effectiveness in combating oxidation [40] when incorporated into liposomes. The findings from this study imply that refrigerated or water bath storage (at temperatures ranging from 4 to 17°C) for 24–48 h may yield superior fertility outcomes compared with frozen semen.

Nevertheless, the impact varied considerably depending on the specific resveratrol types and concentrations. Moreover, methodological diversity was observed across the various studies. This diversity, encompassing differences in the substances examined, their dosages, the sperm parameters assessed, and the AP evaluated, complicates the feasibility of conducting a meta-analysis that could yield more coherent findings. Consequently, future research should explore resveratrol doses with distinct mechanisms of action to develop an optimal preservation medium capable of sustaining long-term boar semen storage while maintaining fertility potential.

From a practical standpoint, incorporating resveratrol into AI programs presents a promising approach to improving reproductive efficiency, particularly in large-scale operations. Resveratrol’s ability to enhance sperm motility, viability, and MI while mitigating oxidative stress could significantly increase the success rates of AI and contribute to genetic improvement in swine populations. However, successful implementation on a commercial scale requires addressing several key factors. The cost-effectiveness of resveratrol supplementation should be assessed relative to conventional antioxidants such as vitamin E and glutathione, given its potential to reduce semen wastage and improve fertility outcomes. Ensuring the availability of standardized extender formulations that effectively incorporate resveratrol is essential for consistent results across storage conditions, including refrigeration and freezing. In addition, logistical factors must be addressed to enable broader adoption, such as the distribution of resveratrol-enriched extenders and the training required for their proper use.

One of the key strengths of this systematic review lies in its comprehensive evaluation of the existing literature, which highlights the potential of resveratrol as a promising antioxidant for boar semen preservation. Including diverse studies from different experimental settings allows a broad understanding of resveratrol’s biological effects, particularly its antioxidant, anti-inflammatory, and anti-apoptotic properties. Moreover, the focus on nano-encapsulation as a delivery method underscores the innovative approaches being explored to enhance antioxidant efficacy during semen preservation. These findings provide a valuable foundation for further experimental validation of preservation media.

However, this study also has several limitations that must be acknowledged. The significant heterogeneity among the included studies, in terms of experimental design, concentrations of resveratrol used, and assessed parameters, limits the ability to perform robust meta-analysis. This variability hampers the generalizability of findings and complicates the derivation of standardized recommendations for practical application. In addition, the lack of direct comparative studies on resveratrol in boar semen under different preservation conditions, such as liquid storage versus cryopreservation, represents a critical research gap. Future investigations should aim to address these limitations by employing standardized methodologies, evaluating dose-response relationships, and exploring long-term storage outcomes to fully elucidate resveratrol’s potential in boar semen preservation.

## CONCLUSION

This systematic review underscores the significant potential of resveratrol as an antioxidant for boar semen preservation, highlighting its beneficial effects under various storage conditions, from bath-cooled to frozen semen. The analysis of existing studies demonstrates that supplementation with resveratrol, particularly within the concentration range of 25–200 μM, consistently improves key quality parameters, including sperm motility, viability, MI, and mitochondrial function, while reducing oxidative stress. These improvements directly enhance the fertility potential of preserved boar semen. Despite these promising findings, further comprehensive studies are needed to validate and expand upon these results. Future research should employ standardized protocols to evaluate the effects of resveratrol across a full spectrum of parameters, such as sperm motility, viability, membrane and AI, DNA integrity, oxidative stress markers (e.g., reactive oxygen species, lipid peroxidation), and antioxidant enzyme activities (e.g., superoxide dismutase, glutathione, catalase). Such studies should also investigate a wider range of dosages to determine the optimal conditions for resveratrol use in boar semen preservation. By identifying the most effective concentrations and storage protocols, it will be possible to maximize the reproductive outcomes of stored boar semen and advance the field of semen preservation in swine reproduction.

## AUTHOR’S CONTRIBUTIONS

RAP: Conceived and designed the study, co-ordinated the research project, and drafted the manuscript. TS: Study design and data collection and analysis. ALS: Literature review, data extraction, and interpretation of the results. SYH: Critical revision of the manuscript, provided valuable insights into the discussion and conclusion, and contributed to the interpretation of data and writing of key sections. SMS: Statistical analysis and drafted and revised the manuscript. All authors have read and approved the final manuscript.

## References

[1] Martín-Hidalgo D, Hurtado De Llera A, Henning H, Wallner U, Waberski D, Bragado M.J, Gil M.C, Garcia-Marin L.J (2013). The effect of resveratrol on the quality of extended boar semen during storage at 17^o^C. J. Agric. Sci.

[2] Sun L, Wu C, Xu J, Zhang S, Dai J, Zhang D (2020). Addition of butylated hydroxytoluene (BHT) in tris-based extender improves post-thaw quality and motion dynamics of dog spermatozoa. Cryobiology.

[3] Wang S, Sun M, Wang N, Yang K, Guo H, Wang J, Zhang Y, Yue S, Zhou J (2018). Effects of L-glutamine on boar sperm quality during liquid storage at 17°C. Anim. Reprod. Sci.

[4] Cerolini S, Maldjian A, Surai P, Noble R (2000). Viability, susceptibility to peroxidation and fatty acid composition of boar semen during liquid storage. Anim. Reprod. Sci.

[5] Kim S, Lee Y.J, Kim Y.J (2011). Changes in sperm membrane and ROS following cryopreservation of liquid boar semen stored at 15°C. Anim. Reprod. Sci.

[6] Giaretta E, Estrada E, Bucci D, Spinaci M, Rodríguez-Gil J.E, Yeste M (2015). Combining reduced glutathione and ascorbic acid has supplementary beneficial effects on boar sperm cryotolerance. Theriogenology.

[7] Breininger E, Descalzo A, Rossetti L, Abramovich D, Beconi M.T (2011). Boar sperm functionality is related to -tocopherol content after freezing-thawing:Antioxidants in boar sperm cryopreservation. Andrologia.

[8] Feugang J.M, Rhoads C.E, Mustapha P.A, Tardif S, Parrish J.J, Willard S.T, Ryan P.L (2019). Treatment of boar sperm with nanoparticles for improved fertility. Theriogenology.

[9] Zhang X, Liu Q, Wang L, Yang G, Hu J (2016). Effects of glutathione on sperm quality during liquid storage in boars. Anim. Sci. J.

[10] Li H, Zhang X, Fang Q, Liu Q, Du R, Yang G, Wang L.Q, Hu J.H (2017). Supplemental effect of different levels of taurine in Modena on boar semen quality during liquid preservation at 17°C. Anim. Sci. J.

[11] Kovacic P, Somanathan R (2010). Multifaceted approach to resveratrol bioactivity:Focus on antioxidant action, cell signaling and safety. Oxid. Med. Cell. Longev.

[12] Athar M, Back J, Tang X, Kim K, Kopelovich L, Bickers D, Kim A.L (2007). Resveratrol:A review of preclinical studies for human cancer prevention. Toxicol. Appl. Pharmacol.

[13] Guerrero R.F, Liazid A, Palma M, Puertas B, González-Barrio R, Gil-Izquierdo Á, García-Barroso C, Cantos-Villar E (2009). Phenolic characterisation of red grapes autochthonous to Andalusia. Food Chem.

[14] Pervaiz S, Holme A.L (2009). Resveratrol:Its biologic targets and functional activity. Antioxid. Redox Signal.

[15] Tvrdá E, Kováčik A, Tušimová E, Massányi P, Lukáč N (2015). Resveratrol offers protection to oxidative stress induced by ferrous ascorbate in bovine spermatozoa. J. Environ. Sci. Health. Part A.

[16] Bucak M.N, Ataman M.B, Başpinar N, Uysal O, Taşpinar M, Bilgili A, Öztürk C, Güngör S, İnanç M.E, Akal E (2015). Lycopene and resveratrol improve post-thaw bull sperm parameters:Sperm motility, mitochondrial activity and DNA integrity. Andrologia.

[17] Li C.Y, Zhao Y.H, Hao H.S, Wang H.Y, Huang J.M, Yan C.L, Du W.H, Pang Y.W, Zhang P.P, Liu Y, Zhu H.B, Zhao X.M (2018). Resveratrol significantly improves the fertilisation capacity of bovine sex-sorted semen by inhibiting apoptosis and lipid peroxidation. Sci. Rep.

[18] Silva E.C.B, Cajueiro J.F.P, Silva S.V, Soares P.C, Guerra M.M.P (2012). Effect of antioxidants resveratrol and quercetin on *in vitro* evaluation of frozen ram sperm. Theriogenology.

[19] Gadani B, Bucci D, Spinaci M, Tamanini C, Galeati G (2017). Resveratrol and Epigallocatechin-3-gallate addition to thawed boar sperm improves *in vitro* fertilization. Theriogenology.

[20] Bucci D, Spinaci M, Yeste M, Mislei B, Gadani B, Prieto Martinez N, Love C, Mari G, Tamanini C, Galeati G (2018). Combined effects of resveratrol and epigallocatechin-3-gallate on post thaw boar sperm and IVF parameters. Theriogenology.

[21] Nashtaei S.M, Nekoonam S, Naji M, Bakhshalizadeh S, Amidi F (2018). Cryoprotective effect of resveratrol on DNA damage and crucial human sperm messenger RNAs, possibly through 5′AMP-activated protein kinase activation. Cell Tissue Banking.

[22] Soares S.L, Brito C.R.C, Anciuti A.N, Gatti N.C, Corcini C.D, Varela A.S, Marques M.G, Fonseca F.N, Komninou E.R, Lucia T (2021). Nanocarried antioxidants in freezing extenders for boar spermatozoa. Andrologia.

[23] Page M.J, McKenzie J.E, Bossuyt P.M, Boutron I, Hoffmann T.C, Mulrow C.D, Shamseer L, Tetzlaff J.M, Akl E.A, Brennan S.E, Chou R, Glanville J, Grimshaw J.M, Hróbjartsson A (2021). The PRISMA 2020 statement:An updated guideline for reporting systematic reviews. BMJ.

[24] Prommi A, Deesamer J, Namlao P, Chanapiwat P, Kaeoket K (2018). Effect of *Lakoocha extracteded* (oxyresveratrol) on the quality of frozen boar semen. J. App. Anim. Sci.

[25] He W, Zhai X, Duan X, Di H (2020). Effect of resveratrol treatment on apoptosis and apoptotic pathways during boar semen freezing. J. Zhejiang Univ. Sci. B.

[26] Torres M.A, Rigo V.H.B, Leal D.F, Pavaneli A.P.P, Muro B.B.D, de Agostini Losano J.D, Kawai G.K.V, Collado M.D, Perecin F, Nichi M, Martins S.M.M.K, de Andrade A.F.C (2021). The use of resveratrol decreases liquid-extend boar semen fertility, even in concentrations that do not alter semen quality. Res. Vet. Sci.

[27] Zhu Z, Li R, Fan X, Lv Y, Zheng Y, Hoque S.M, Wu D, Zeng W (2019). Resveratrol improves boar sperm quality via 5′AMP-activated protein kinase activation during cryopreservation. Oxid. Med. Cell. Longev.

[28] Kaeoket K, Chanapiwat P (2023). The beneficial effect of resveratrol on the quality of frozen-thawed boar sperm. Animals (Basel).

[29] Yang K, Wang N, Guo H.T, Wang J.R, Sun H.H, Sun L.Z, Yue S.L, Zhou J.B (2020). Effect of L-carnitine on sperm quality during liquid storage of boar semen. Asian-Australasian J. Anim. Sci.

[30] Silvestre M.A, Yániz J.L, Peña F.J, Santolaria P, Castelló-Ruiz M (2021). Role of antioxidants in cooled liquid storage of mammal spermatozoa. Antioxidants (Basel).

[31] Ammar O, Houas Z, Mehdi M (2019). The association between iron, calcium, and oxidative stress in seminal plasma and sperm quality. Environ. Sci. Pollut. Res.

[32] Serrano R, Garrido N, Céspedes J.A, González-Fernández L, García-Marín L.J, Bragado M.J (2020). Molecular mechanisms involved in the impairment of boar sperm motility by peroxynitrite-induced nitrosative stress. Int. J. Mol. Sci.

[33] Longobardi V, Salzano A, Campanile G, Marrone R, Palumbo F, Vitiello M, Zullo G, Gasparrini B (2017). Carnitine supplementation decreases capacitation-like changes of frozen-thawed buffalo spermatozoa. Theriogenology.

[34] Hussein R, Abbas L, Rayhaan S, Fadhil H, AL-Mousawi Z (2023). The impact of adding melatonin and other antioxidants on post-thaw human sperm quality during cryopreservation. Med. J. Babylon.

[35] Altyeb Y.H, Absy G, Medan M.S, Hassan S.T, Elsayed D.H (2022). The effects of cysteine and L-carnitine on the DNA integrity of post-thaw sperm of frozen buck semen. Iranian J. Vet. Res.

[36] Rezaei A, Bahmani H.R, Mafakheri S, Farshad A, Nazari P (2022). Protective effects of different doses of MitoQ separately and combined with trehalose on sperm function and antioxidative status of cryopreserved Markhoz goat semen. Cryobiology.

[37] Balamurugan B, Ghosh S.K, Lone S.A, Prasad J.K, Ramamoorthy M, Kumar A (2022). Correlation between dissolved oxygen level, antioxidants and oxidants in semen diluted with partially deoxygenated extender at various stages of cryopreservation. Cryo Letters.

[38] Hu Q (2023). Effects of zinc chloride on boar sperm quality during liquid storage at 17°C. Vet. Med. Sci.

[39] Najafi A, Daghigh Kia H, Hamishehkar H, Moghaddam G, Alijani S (2019). Effect of resveratrol-loaded nanostructured lipid carriers supplementation in cryopreservation medium on post-thawed sperm quality and fertility of roosters. Anim. Reprod. Sci.

[40] Suntres Z.E (2011). Liposomal antioxidants for protection against oxidant-induced damage. J. Toxicol.

